# Contraceptive use following spontaneous and induced abortion and its association with family planning services in primary health care: results from a Brazilian longitudinal study

**DOI:** 10.1186/s12978-015-0087-7

**Published:** 2015-10-15

**Authors:** Ana Luiza Vilela Borges, Funmilola OlaOlorun, Elizabeth Fujimori, Luiza Akiko Komura Hoga, Amy Ong Tsui

**Affiliations:** Department of Public Health Nursing, University of São Paulo School of Nursing, São Paulo, Brazil; Department of Preventive Medicine and Primary Care, University of Ibadan, Ibadan, Nigeria; Department of Maternal, Child and Psychiatric Care, University of São Paulo School of Nursing, São Paulo, Brazil; Department of Population, Family and Reproductive Health, Johns Hopkins Bloomberg School of Public Health, Baltimore, USA; School of Nursing, University of São Paulo, Av. Dr. Enéas Carvalho de Aguiar, 419, cep 05403-000 São Paulo, Brazil

**Keywords:** Sexual and reproductive health, Post-abortion, Family planning, Pregnancy intention, Abortion

## Abstract

**Background:**

Although it is well known that post-abortion contraceptive use is high when family planning services are provided following spontaneous or induced abortions, this relationship remains unclear in Brazil and similar settings with restrictive abortion laws. Our study aims to assess whether contraceptive use is associated with access to family planning services in the six-month period post-abortion, in a setting where laws towards abortion are highly restrictive.

**Methods:**

This prospective cohort study recruited 147 women hospitalized for emergency treatment following spontaneous or induced abortion in Brazil. These women were then followed up for six months (761 observations). Women responded to monthly telephone interviews about contraceptive use and the utilization of family planning services (measured by the utilization of medical consultation and receipt of contraceptive counseling). Generalized Estimating Equations were used to analyze the effect of family planning services and other covariates on contraceptive use over the six-month period post-abortion.

**Results:**

Women who reported utilization of both medical consultation and contraceptive counseling in the same month had higher odds of reporting contraceptive use during the six-month period post-abortion, when compared with those who did not use these family planning services [adjusted aOR = 1.93, 95 % Confidence Interval: 1.13–3.30]. Accessing either service alone did not contribute to contraceptive use. Age (25–34 vs. 15–24 years) was also statistically associated with contraceptive use. Pregnancy planning status, desire to have more children and education did not contribute to contraceptive use.

**Conclusions:**

In restrictive abortion settings, family planning services offered in the six-month post-abortion period contribute to contraceptive use, if not restricted to simple counseling. Medical consultation, in the absence of contraceptive counseling, makes no difference. Immediate initiation of a contraceptive that suits women’s pregnancy intention following an abortion is recommended, as well as a wide range of contraceptive methods, including long-acting reversible methods, even in restrictive abortion laws contexts.

## Background

Global abortion rates remained stable between 2003 and 2008, but estimates for Latin American countries showed a slight decrease [1]. Although this appears to be a favorable development, the region continues to contend with restrictive laws regarding induced abortion. This means the majority of abortions occur under relatively unsafe conditions. Taking Brazil as an example,—the setting for this study-induced abortion is legal only in situations of sexual violence, risk to the woman’s life and fetal anencephaly. Although the country has seen reduced economic inequalities and improved the organization of its health services [2], abortion is still a priority health concern. One of the reasons abortion remains important is the magnitude of the problem. In 2010, 22 % of women aged 35–39 years reported having had at least one induced abortion during their lifetime [3]. Nearly 200,000 women ages 15–49 are hospitalized every year due to consequences of induced and spontaneous abortion [4], and the attendant high maternal mortality ratios continue to prevent Brazil from attaining the fifth Millennium Development Goal [2], which is concerned with the improvement of maternal health, by reducing maternal mortality ratio, and by achieving universal access to reproductive health [5].

As with many other women from countries with restrictive abortion laws, Brazilian women who desire to terminate a pregnancy usually seek care in hospitals following self-induction with misoprostol [6–8]. Women who have spontaneous abortions may experience the same medical complications observed with abortions induced with misoprostol, thereby also needing emergency treatment. To address this issue, the Brazilian Ministry of Health revised The National Technical Guidelines for Abortion Care in 2011, based on the guidelines by the World Health Organization and International Federation of Gynecology and Obstetrics [9, 10]. The aim of the revision was to improve the quality of care provided to all women with pregnancy loss, irrespective of their abortion status [11]. The guidelines include technical recommendations on the basic components of post-abortion care (PAC): emergency treatment for complications of spontaneous or induced abortions, offer of contraceptive counseling and methods, and referral to other reproductive health services [12]. It also recommends social and mental support to women in this situation.

However, the stigma associated with illegal abortion can compromise the quality of health care in Brazil and elsewhere [7, 8, 13, 14]. In a multicountry study, authors observed that abortion is a highly stigmatized behavior. Thus, when seeking health care, women first tend to report that the abortion is spontaneous, and then later, might admit they attempted to terminate the pregnancy by themselves [15]. This strategy to avoid punishments while trying to obtain health care has also been observed in the Caribbean islands and Chile [13, 16]. In settings with restrictive abortion laws, women with spontaneous abortion may also experience stigmatization as they access the same health services [17].

In Brazil, the Unified Health System, or SUS, is based on the principles of health as a universal right to be fulfilled by the state. Its aim is to provide universal care through a decentralized management system with specific duties for municipalities, states and the federal union, as well as primary, secondary and tertiary health services, providing care for 75 % of the population. Private services and providers are complementary to SUS [2, 18].

Women who can afford to pay for private services can access safe abortion, though still illegal. Lower income women, on the other hand, tend to use misoprostol and seek for emergency treatment at public hospitals. However, misoprostol commerce is forbidden in the country, so women have to purchase it from the black market, thus highlighting women’s health and social risks while trying to terminate a pregnancy in such restrictive contexts. Legal abortions are provided only at SUS [8, 18].

Brazilian guidelines also consider that family planning counseling and provision of contraceptive methods are key components of post-abortion care. This recommendation is based on the fact that following pregnancy termination and resumption of sexual activity, women are at risk of becoming pregnant again as early as two weeks after the procedure [10], even though a pregnancy interval of at least six months after any abortion (spontaneous or induced) is recommended [19]. This is supported by the evidence that an inter-pregnancy interval shorter than six months increases the risk of preterm births, low birth weight, premature rupture of membranes and spontaneous abortion [19, 20].

Contraceptive acceptance and use are high when post-abortion family planning services are provided [21, 22], but this is far from reality for the majority of women in countries with restrictive laws toward induced abortion [23]. While emergency treatment may be delivered at satisfactory levels in some restrictive abortion countries [14, 17], the full package of family planning counseling, education and methods are often not provided before the woman leaves the health care facility where she received abortion care, as recommended [24]. In Nigeria, for instance, 20 % of post-abortion women received family planning counseling, whereas only 3 % received contraceptive methods [6]. In Ethiopia, the proportion that received contraceptive methods was 56 % [25]. In Sri Lanka, researchers have observed that existing post-abortion care is deficient in education and family planning services [14]. In Brazil, the only observational study that assessed the quality of post-abortion care suggests that the situation may even be worse in this country, as only 7.9 % of women received a prescription for a contraceptive before hospital discharge [17].

For women who are unable to obtain family planning services, including counseling during routine post-abortion hospital care, linkages to other reproductive health services should be provided. In Brazil, this path should be achieved by the hospital referring women to the SUS primary health care level [8, 18], where the national family planning program is implemented. The Brazilian family planning program provides free contraceptive methods in primary health care facilities. Hormonal methods and the copper Intra Uterine Device (IUD) are available only with medical prescription; female and male sterilization candidates are first interviewed and counseled, and then referred to a SUS secondary level institution after completing formal requirements, e.g., two counseling sessions and a psychological assessment. Implants, hormonal IUD, patches and vaginal rings are not available at primary health care facilities. If a woman/couple desires a barrier method, such as a condom or diaphragm, she/they can get it directly from the primary health care facility pharmacy without consulting a health provider.

Although the national family planning program was first implemented in 1996, women and couples still face some barriers when trying to access contraceptive methods in primary health care clinics, like limited contraceptive choice, few trained providers to insert IUD, having to make multiple visits to the clinic to obtain a method, long waiting time to access a medical consultation in order to obtain hormonal contraception, and poor integration with other existing health services [26–28]. Due to these barriers, oral pill is commonly bought directly from drugstores, even without medical prescription. Even though it is not formally assigned, this is common practice [29]. Women also have the option to access private services when they can afford it.

Since the Brazilian scenario suggests that not every woman who seeks emergency treatment post-abortion receives contraceptive counseling and methods before she leaves the health care facility, it is important to better understand how women in abortion restrictive settings manage to prevent a pregnancy in the 6 months after being treated for abortion complications. Considering the gap in the full understanding of how to increase contraceptive protection following an abortion in such contexts, our study aims to assess whether contraceptive use is associated with access to family planning services in the six-month period following a pregnancy termination in a setting where laws toward abortion are highly restrictive.

## Methods

This 6-month prospective cohort study was conducted on a sample of women recruited while hospitalized for emergency treatment following either an induced or spontaneous abortion in a SUS maternity hospital in Sao Paulo, Brazil. The institution is located in the central area of the city and serves as a referral center for women with high-risk pregnancies, delivering care to about 450 births and 30 post-abortion women every month. Women who seek emergency treatment care after a pregnancy termination generally remain hospitalized for no more than 24 h per institutional routine. The institution follows the National Guidelines for Abortion Care [11] and usually sets a follow-up appointment for the 45th day after discharge.

### Study population

**Fig. 1:**
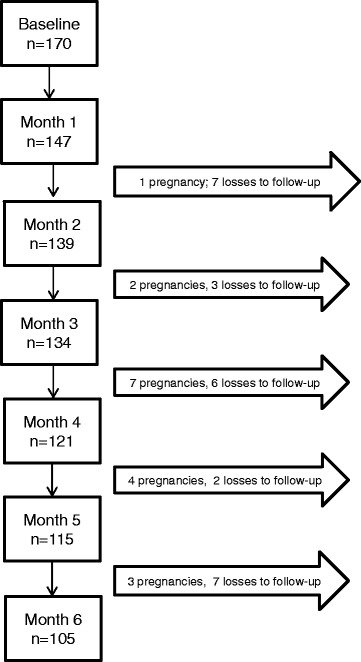
Number of women participating in follow-up over the six month period of the study

The purposive sample included all women hospitalized following an abortion at this SUS maternity, between May and December, 2011, irrespective of whether their abortion was spontaneous or induced (*n* = 184). Exclusion criteria included poor understanding of Portuguese language (*n* = 6), not having a telephone for study contact (*n* = 0), and being hospitalized for legal abortion (*n* = 0). Eight women refused. Those who consented (*n* = 170) answered a structured questionnaire at baseline. After the first month, 147 women were traced (82 %) and constituted the study cohort. Losses over this period were due to 8 refusals, 1 maternal death and 14 women who could not be located. Women in the cohort were contacted by telephone every 30 days for 6 months. Once women reported a confirmed pregnancy in response to the questions “*Are you pregnant? If yes, have you confirmed it with a pregnancy test?*”, they were excluded from the study. The last telephone interview was conducted in August 2012. Overall, 17 % of women traced at the first post-abortion month were lost to follow up by the sixth month; hence 105 women completed all six interviews (71 %). The 17 % lost to follow-up (*n* = 25) were the result of changes in telephone numbers and two refusals (Fig. 1).

### Study instruments and data collection

Two questionnaires were used. The first, administered by trained midwives through a face-to-face baseline interview during the period of hospitalization, collected data on social and demographic characteristics and reproductive history. Due to legal restrictions on abortion, it was expected that the reason for seeking the procedure would be inaccurately reported and therefore underestimated. Thus, the reason was not asked of women. To assess whether the terminated pregnancy was unplanned or planned, we used the Brazilian Portuguese validated version [30] of the London Measure of Unplanned Pregnancy (LMUP) [31]. The second questionnaire was administered by trained social researchers by telephone one month after discharge and every 30 days for 6 months (Months 1–6); it gathered data on contraceptive use, utilization of primary health care services, future pregnancy intention and pregnancy occurrence.

The outcome of interest was contraceptive use over the 6-month period following the abortion (dichotomous variable with no/yes categories).

The main independent variable was utilization of family planning services 6 months following the abortion. We created this variable based on responses received during the monthly telephone interviews over the six-month period following hospital discharge, so this information refers to the 30-day period preceding each monthly interview. As family planning services are delivered at the primary health care level in Brazil (exceptions are female and male sterilization), but women are free to obtain services from private providers, we assessed the utilization of two types of services: medical consultation and contraceptive counseling, which allowed us to collect data on a combination of ways to obtaining a contraceptive.

We used medical consultation since doctors are the only health professionals who are currently allowed to prescribe hormonal contraception and trained to insert IUDs in Brazil. The exception is emergency contraception, which nurses can also prescribe. Thus, users of public health facilities who seek non-barrier contraceptive methods must make an appointment to see a doctor for a prescription, as in the private sector.

We also measured contraceptive counseling receipt separately because this is carried out, both individually and in groups, by a range of other health professionals, e.g., nurses, pharmacists, nurse auxiliaries, community health agents, and social workers, and can take place apart from medical consultation, such as during home visiting, health promotion and educational meetings, and purchases at drugstores. Contraceptive counseling was measured through two questions: whether women received information about contraception from a healthcare worker in the 30 days preceding the interview (no/yes), and whether they received information from a healthcare worker in the 30 days preceding the interview regarding risks of inter-pregnancy intervals less than 6 months after a pregnancy loss (no/yes). A positive answer in either question was counted as receipt of contraceptive counseling.

To sum up, we asked women every month if (a) they had a medical consultation in the preceding 30-day period (no/yes); and (b) they received any contraceptive counseling in the preceding 30-day period (no/yes). The variable generated consisted of four categories: (1) report of neither medical consultation nor contraceptive counseling in the preceding 30-day period (reference); (2) only contraceptive counseling in the preceding 30-day period; (3) only medical consultation in the preceding 30-day period; and (4) report of both contraceptive counseling and medical consultation in the preceding 30-day period.

In this study, we assume that effective contraceptive services include a combination of being able to simultaneously utilize contraceptive counseling and medical consultation, irrespective of whether women utilized private services, or accessed primary health care facilities or drugstores.

Other covariates were pregnancy planning status (baseline; categorized as unplanned, ambivalent and planned); desire to have more children (baseline; categorized as no/yes); age (baseline; categorized as 15–24, 25–34, and 35–44 years); and education (baseline; measured in completed years of schooling).

For sample description purposes, we collected and report data on women’s age (mean), age at first intercourse (mean), age at first pregnancy (mean), number of previous pregnancies (mean), race/ethnic color (self-identified as white, brown, or black), religion (categorized as Catholic, Protestant, Others and None), work status/employment (no/yes), cohabitation with partner (no/yes), pregnancy planning status (classified from LMUP as planned, ambivalent and unplanned), and sexual activity in the preceding month (no/yes). The type of contraceptive method used is reported for the first and sixth month (no use, pill, condom, injectable, IUD, and traditional, the latter referring to withdrawal and rhythm). We also describe if women were provided with a contraceptive prescription during hospitalization. This variable was based on responses to the question “Did you receive any prescription of contraceptive while hospitalized for emergency treatment following the pregnancy loss?”, which was asked during the telephone interview that occurred 30 days after hospital discharge.

We obtained administrative approval to conduct the study from the University of Sao Paulo School of Nursing and ethical approval from the Maternity Research Ethics Committee. Informed written consent was obtained from all the participants. During recruitment, we emphasized that women could withdraw from the study at any time, with no effect on their health care. In order to protect women’s safety and confidentiality during the telephone interviews, we set a schedule together, based on the most appropriate time and day of the week to call them. We also agreed about what to say when we called them, who we could talk to and if we could identify ourselves as researchers, or as another person/institution. Telephone numbers were noted as being unique or shared. In order to ensure we were talking to the participant herself, we would use check questions, based on information obtained in the previous interview.

### Statistical analysis

The analytic sample consisted of women who completed the baseline interview and were traced at one month post-abortion (*n* = 147). Double data entry by two different operators was done using Epi Info version 6.04. Questionable entries were reconciled.

We describe sociodemographic characteristics of all participants at two time points: in the first post-abortion month (Month 1) and in the sixth post-abortion month (Month 6), as well as the characteristics of those who used contraception. We also describe women who reported a confirmed pregnancy anytime during follow-up. Additionally, we describe the degree of medical consultation utilization and contraceptive counseling receipt each month.

As this is a longitudinal study, we need to account for repeated and correlated observations. Therefore, we used Generalized Estimating Equations (GEE) considering an unstructured correlation matrix and logit link transformation function. GEE is a non-likelihood based method utilized for marginal models of non-linear responses. It is used in longitudinal data when the focus is the difference in the population-average response between two groups with different risk factors [32]. The dependent variable was contraceptive use over the six-month post-abortion period; the main independent variable was utilization of family planning services 6 months post-abortion; and covariates were pregnancy planning status, desire to have more children, age, and education. The multivariate analysis was adjusted for time (i.e., observation for six months) and did not include women who were provided with a contraceptive prescription during hospitalization. Crude and adjusted odds ratios and 95 % confidence intervals were estimated. All analyses were conducted using Stata 13.0.

## Results

For the initial cohort of 147 women, we have observations on a total of 761 person-months over the 6-month study period. Table 1 shows that women in Month 1 were 29.1 (sd = 7.4) years old on average, and had a mean of 9.3 (sd = 2.5) years of schooling. Almost half of them self-identified as white and the majority were Catholic or Protestant. Nearly 60.0 % were working. Two thirds (66.7 %) reported they wanted more children. Women at Month 1 and those who remained in the study by Month 6 did not show any statistically significant difference in their sociodemographic and reproductive characteristics (data not shown).

**Table 1:** Demographic characteristics of all participants, of participants who used contraception, and of participants who reported a pregnancy. Sao Paulo, 2012

Variables	All participants		Participants who used contraception		Participants who reported pregnancy
	Time Point		Time Point		Time Point
	Month 1	Month 6	Month 1	Month 6	Endline
Mean age (years)	29.1 (7.4)	29.6 (7.3)	29.5 (6.8)	29.7 (7.4)	26.8 (6.9)
Mean age at first intercourse (years)	17.2 (2.9)	17.4 (2.9)	17.2 (3.1)	17.4 (2.8)	16.9 (1.9)
Mean age at first pregnancy (years)	21.7 (5.2)	21.9 (5.1)	22.0 (4.9)	21.7 (5.0)	20.7 (5.1)
Mean number of previous pregnancies	2.5 (1.4)	2.5 (1.4)	2.6 (1.4)	2.6 (1.5)	2.3 (1.1)
Mean educational level (years)	9.3 (2.5)	9.5 (2.5)	9.5 (2.6)	9.3 (2.6)	9.8 (1.4)
Color (%)					
White	49.6	55.2	50.6	52.9	47.1
Brown	45.6	40.0	47.1	44.7	52.9
Black	4.8	4.8	2.3	2.4	-
Religion (%)					
Catholic	43.5	43.8	46.0	45.9	35.4
Protestant	32.7	35.2	31.0	34.1	29.4
Other	6.1	5.7	6.9	5.9	17.6
None	17.7	15.3	16.1	14.1	17.6
Employed (%)	59.9	60.9	64.4	58.8	58.8
Current living with partner (%)	76.9	76.2	82.8	74.1	88.2
Desire to have more children (%)	66.7	66.7	70.1	66.5	76.5
Previous pregnancy plan (%)					
Planned	29.9	31.4	29.9	27.1	41.2
Ambivalent	49.0	49.5	50.6	50.6	35.3
Unplanned	21.1	19.1	19.5	22.3	23.5
Total number of women	147	105^a^	87	85	17

Standard deviation (sd) in parentheses

^a^not included 17 women who reported a pregnancy

**Fig. 2:**
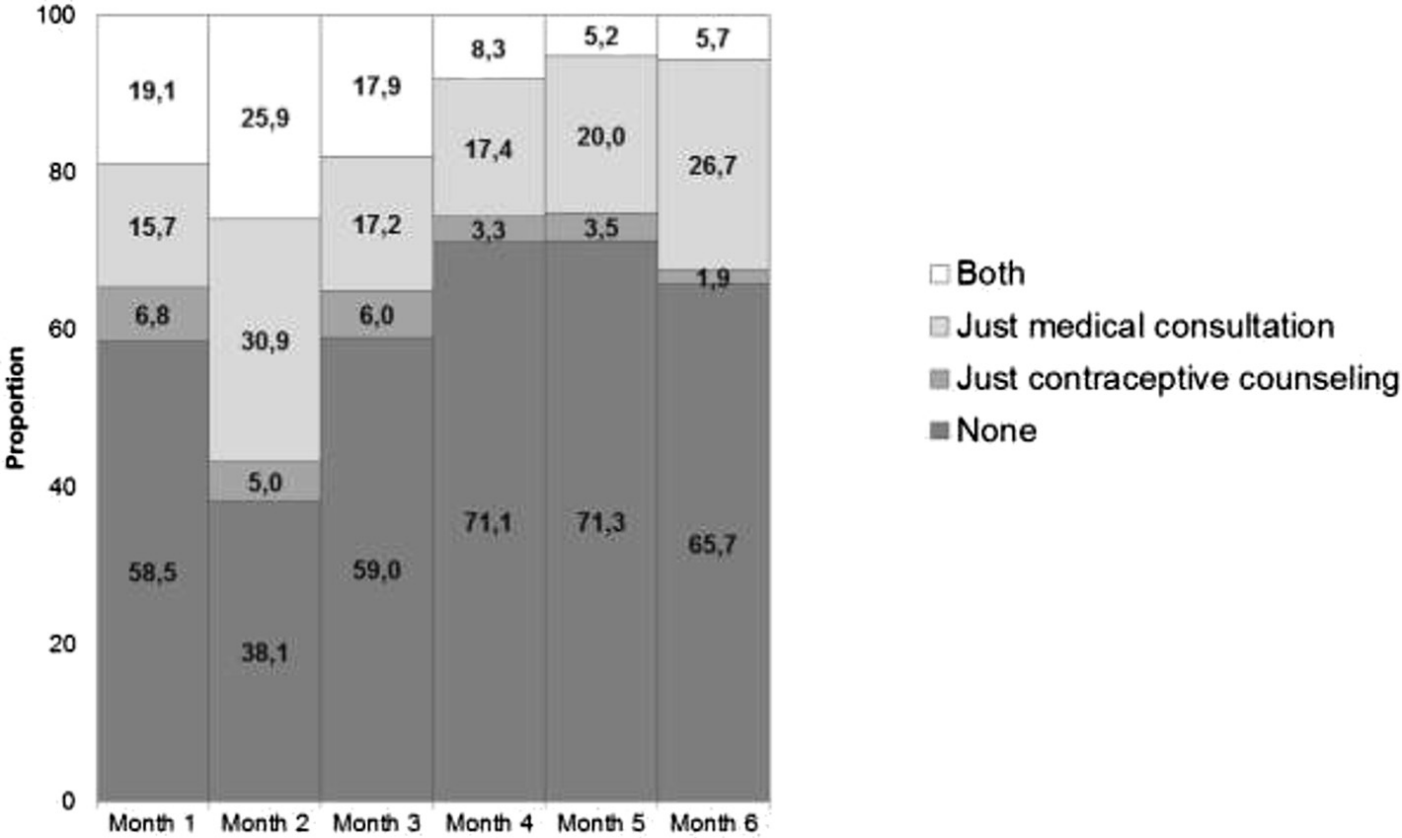
Utilization of medical consultation and receipt of contraception counseling reported by study respondents over a 6-month period post-abortion, by month. Sao Paulo, 2012

Only 8.8 % of clients were provided with a contraceptive prescription during hospitalization. Figure 2 shows utilization of medical consultation and contraceptive counseling receipt from Month 1 to Month 6. More than half of the women did not report access to either medical consultation or contraceptive counseling over the study period, with the exception of Month 2. Medical consultation utilization and contraceptive counseling receipt in the same month had its peak in the second month and sharply decreased thereafter.

**Fig. 3:**
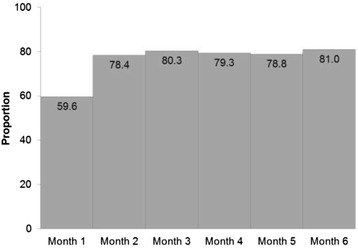
Percent of study respondents using contraception over the six-month post-abortion period, by month. Sao Paulo, 2012

Sexual activity resumed for most women soon after the abortion (69.3 % at Month 1). By Month 6, the majority of women (95.2 %) reported sexual relations in the preceding 30 days. Prevalence of contraceptive use was 59.2 % and 80.9 % at the first and sixth months, respectively (Fig. 3). The main methods reported by study respondents at the first month were condom (39.4 %) and oral pill (14.9 %), while the most reported method at the end of the study was oral pill (35.2 %). Use of injectables also increased from 3.4 to 11.4 % over the study period, but the reported use of condoms decreased to 26.7 %. Use of traditional methods was rare, but steadily increased over time, reported by 3.2 % (Month 1) to 5.8 % (Month 6) of respondents. Whereas reports on IUD use varied from 0.7 to 1.5 % in the six months of follow-up, no women reported use of implants, vaginal rings, patches or sterilization.

The results of analyzing the longitudinal data with GEE methods are shown in Table 2. Pregnancy planning status, desire to have more children, and years of schooling were not associated with the use of contraceptive methods six months following an abortion. On average, women who reported receipt of both medical consultation and contraceptive counseling in the same month had significantly higher odds of contraceptive use over the six month post-abortion period, when compared with those who reported no exposure to either of these services [adjusted OR, aOR = 1.93, 95 % CI: 1.13–3.30]. Receiving either medical consultation or contraceptive counseling alone in the same month did not significantly contribute to the likelihood of contraceptive use. This finding is consistent both in univariate and multivariate analysis. Women 25–34 years old had higher odds of contraceptive use compared with their younger counterparts.

**Table 2:** Number and proportion of women who used contraceptive method and crude and adjusted odds ratios and 95 % confidence intervals showing influence of access to family planning services, and sociodemographic

Variables	Contraceptive use in the 6 months following an abortion						Univariate analysis		Multivariate analysis	
	Month 1	Month 2	Month 3	Month 4	Month 5	Month 6	OR_crude_	95 % CI	OR_adjusted_^a^	95 % CI
Utilization of family planning services in the previous month										
None	52 (59.8 %)	39 (35.7 %)	60 (56.1 %)	65 (67.7 %)	65 (73.0 %)	60 (70.6 %)	1.00	-	1.00	-
Just contraceptive counseling	4 (4.6 %)	6 (5.5 %)	7 (6.5 %)	3 (3.1 %)	4 (4.5 %)	2 (2.3 %)	1.05	0.51–2.15	1.16	0.56–2.38
Just medical consultation	12 (13.8 %)	32 (29.4 %)	18 (16.8 %)	18 (18.7 %)	15 (16.9 %)	19 (22.4 %)	0.88	0.60–1.30	0.91	0.60–1.38
Both	19 (21.8 %)	32 (29.4 %)	22 (20.6 %)	10 (10.4 %)	5 (5.6 %)	4 (4.7 %)	1.93	1.17–3.18	1.93	1.13–3.30
Pregnancy planning status										
Unplanned	17 (19.5 %)	23 (21.1 %)	25 (23.4 %)	23 (24.0 %)	16 (18.0 %)	19 (22.3 %)	1.00	-	1.00	-
Ambivalent	44 (50.6 %)	54 (49.5 %)	50 (46.7 %)	43 (44.8 %)	49 (55.1 %)	43 (50.6 %)	0.91	0.49–1.69	0.79	0.43–1.46
Planned	26 (29.9 %)	32 (29.4 %)	32 (29.9 %)	30 (31.2 %)	24 (26.9 %)	23 (27.1 %)	0.79	0.40–1.56	0.71	0.36–1.40
Desire to have more children										
No	32 (36.8 %)	50 (45.9 %)	47 (43.9 %)	41 (42.7 %)	46 (51.7 %)	39 (45.9 %)	1.00	-	1.00	-
Yes	55 (63.2 %)	59 (54.1 %)	60 (56.1 %)	55 (57.3 %)	43 (48.3 %)	46 (54.1 %)	0.77	0.55–1.08	0.81	0.57–1.15
Age										
15–24 years	19 (21.8 %)	26 (23.8 %)	27 (25.3 %)	27 (28.1 %)	25 (28.1 %)	24 (28.2 %)	1.00	-	1.00	-
25–34 years	44 (50.6 %)	55 (50.5 %)	53 (49.5 %)	45 (46.9 %)	42 (47.2 %)	36 (42.4 %)	2.76	1.53–4.98	2.96	1.61–5.42
35–44 years	24 (27.6 %)	28 (25.7 %)	27 (25.2 %)	24 (25.0 %)	22 (24.7 %)	25 (29.4 %)	1.59	0.86–2.92	1.50	0.78–2.87
Education							1.01	0.92–1.11	1.03	0.93–1.13
Intercept									0.99	0.30–3.25

Odds ratios estimated using Generalized Estimating Equations with uncorrelated matrix and logit link function assumed

^a^Adjusted also by time of observation (1–6 months)

Seventeen women reported a confirmed pregnancy over the 6-month study period, which translates into a pregnancy rate of 115.6 per 1000 women. Table 1 shows that women who became pregnant were younger and reported a higher proportion of previous planned pregnancies than all women in the study. Ten of them, however, reported the new pregnancy was mistimed.

## Discussion

This is the first longitudinal study from Brazil to assess contraceptive use for 6 months subsequent to induced and spontaneous abortion. Loss to follow-up in this cohort was lower than those measured in other longitudinal studies conducted on abortion [21, 33]. Moreover, loss to follow up was at random, as we did not find any significant differences between the characteristics of women at Month 1 and those at endline.

Because we relied on a purposive sample of recruited patients at a particular hospital and point in time, the results of our study cannot be generalized to all post-abortion Brazilian women. Furthermore, it is estimated that only one out of five women obtaining an induced abortion seeks post-abortion care in Brazil [4]. A limitation of our study is that we are unable to determine the extent to which contraceptive use may differ between women with an induced versus spontaneous abortion. The use of a validated measure of pregnancy planning status may have minimized this bias. We also cannot verify if all the information reported over the phone is in fact true.

On the other hand, as a prospective study, these findings enable making guarded causal inferences to explain the relationship between contraceptive services and post-abortion reproductive behavior with a perspective from health service delivery at the primary health care level. At the same time, the study setting in Brazil offers a very specific context of restrictive laws regarding abortion, which may also be applicable to almost half of the countries in the world [34], not just Latin America.

Our study shows that post-abortion family planning care is poorly delivered. In fact, very few women were provided with contraceptive prescription while hospitalized, just as was observed by Aquino et al. in three Brazilian capitals [17]. In a review study about PAC initiatives in Latin America [35], other researchers have pointed out the reasons for the lack of contraceptive services during the hospitalization included “the physical and administrative separation between the ward or area where emergency treatment services are provided and on-site family planning clinics, limited accessibility to contraceptive methods for staff not working in the family planning clinic, and minimal staff knowledge about post-abortion contraception”. Regardless of the reasons why post-abortion delivery of contraceptives was so low, PAC should not focus only on the treatment of complications for incomplete abortion, but also on preventing repeat unintended pregnancy.

Except for the second month post-abortion, more than half of women reported having received neither contraceptive counseling nor medical consultation. The second post-abortion month was the period when women could most easily utilize medical consultation, with or without contraceptive counseling. The appointment set by the maternity hospital for the 45^th^ day post-abortion might have contributed to this peak. On the other hand, women frequently do not return for referral appointments [35] and 45 days after the hospital discharge seems to be late for contraceptive counseling as women resume sexual intercourse earlier.

In considering the family planning services offered at the primary health care level that women can access post-abortion, utilization of both medical consultation and contraceptive counseling in the same month had a positive effect on contraceptive use, but there was no effect when one happened in the absence of the other. It means that seeing a physician does not necessarily lead to contraceptive counseling or to method provision. Studies emphasize that only counseling or simply offering additional methods may not be sufficient to improve contraceptive use among post-abortion women [33, 36]. Effective post-abortion family planning also requires immediate offer of contraceptive methods [22, 37].

Study results should also be interpreted against the backdrop of restrictive abortion laws since abortion issues remain highly stigmatized in Brazilian society. Health professionals do not include abortion conversations during consultations and women often omit this information when interacting with providers. Furthermore, initiatives around prenatal care have priority in primary health care services over family planning [28].

In the first post-abortion month, almost 40 % were unprotected and thus vulnerable to a pregnancy. This finding is particularly important since it has been suggested that 91 % of post-abortion women ovulate within a month after the procedure [38]. Use of contraception was close to 80 % of study participants from the second month and beyond, a proportion similar to the Brazilian 2006 contraceptive prevalence rate (80.6 % among 15–49 year old married women) [29]. This high prevalence of contraceptive use does not apparently translate into effective reproductive planning. A Ministry of Health report reveals that women who undergo an abortion predominantly used contraceptive methods but report practice to be inconsistent or erroneous [39], a finding that reinforces the need to improve the quality of family planning care.

The contraceptive method mix in this study for reversible methods was very similar to the Brazilian profile, with high proportions of condom and pill use reported [29]. Condoms are freely available in primary health care facilities in the country and women can easily obtain them, with no requirement to see a doctor or other health professional. Although pills are generally available through medical prescription, they can also be bought at any drugstore at low prices without prescription, making them very popular. Long-acting reversible contraception (LARC), on the other hand, is quite rare. Even though a LARC method is more likely to be chosen over other methods, if made available at the time of abortion [40] to prevent repeat abortion [41], these methods were infrequently reported in our study, probably reflecting women’s limited access to different methods, rather than their personal choices from a range of contraceptives. The fact that LARC methods are not uniformly offered by the public health system and that trained health professionals are required to insert IUDs may also work as a barrier for their use among Brazilian women. Surprisingly, there were no reports of female sterilization, which is the second most used contraceptive in Brazil (25.9 %) [29]. This may be because women in this study were relatively young, had not completed their families, and had no reason to seek a permanent method of contraception. Also, some women with spontaneous abortion would be prone to get pregnant soon, making permanent methods impracticable. Hormonal injectables showed a slight increase, coinciding with what has been observed over the last decade in the country [29].

Our results have important implications for reproductive health policies as they provide evidence that the presence of national guidelines do not necessarily lead to an adequate utilization of family planning services at the primary health care level, especially if restrictive abortion laws mediate health care delivery. Considering the evidence supporting the importance of early family planning counseling and provision of contraceptive methods on contraceptive use after an abortion, practices in Brazilian public hospitals and primary health care services should change accordingly. In a setting with restrictive legislation around pregnancy termination, it is clear that immediate initiation of a contraceptive that suits women’s pregnancy intentions (especially LARC for those who do not intend to become pregnant soon), along with monitoring their health needs at the first month post-abortion will improve levels of sexual and reproductive health [24]. Our study shows that being able to utilize both medical consultation and contraceptive counseling can contribute to contraceptive use in the six-month period following an abortion, if they occur together. Improving the quality of care to post-abortion women will require implementing task shifting in contraceptive care in order to remove barriers to the access of all types of contraceptives, including LARC, as well as expanding contraceptive choice in primary health care. The occurrence of pregnancies among the study participants within post-abortion intervals of less than six months and one maternal death emphasize the critical nature of this agenda for change in policies and regulations. Substantial efforts remain needed to align women’s reproductive intentions with the best clinical management in Brazil.

## Abbreviations

PAC: Post-abortion care
SUS: Unified health system
IUD: Intra uterine device
LMUP: London measure of unplanned pregnancy
GEE: Generalized estimating equations
